# Screening for fecal carriage of MCR-producing *Enterobacteriaceae* in healthy humans and primary care patients

**DOI:** 10.1186/s13756-017-0186-z

**Published:** 2017-03-14

**Authors:** Katrin Zurfluh, Roger Stephan, Andreas Widmer, Laurent Poirel, Patrice Nordmann, Hans-Jakob Nüesch, Herbert Hächler, Magdalena Nüesch-Inderbinen

**Affiliations:** 1 Institute for Food Safety and Hygiene, Vetsuisse Faculty University of Zurich, Winterthurerstrasse 272, 8057 Zurich, Switzerland; 2 Division of Infectious Diseases and Hospital Epidemiology, University of Basel, 4031 Basel, Switzerland; 3 Emerging Antibiotic Resistance, Medical and Molecular Microbiology Unit, Department of Medicine, University of Fribourg, 1700 Fribourg, Switzerland; 4 Practice for General Medicine, Birchstrasse 2, 8472 Seuzach, Switzerland

**Keywords:** Colistin, MCR, Fecal carriage, Population

## Abstract

**Background:**

The extent of the occurrence of the plasmid-encoded colistin resistance genes *mcr-1* and *mcr-2* among humans is currently sparsely studied in Western Europe.

**Objectives:**

To determine the occurrence of MCR-producing *Enterobacteriaceae* in fecal samples of healthy humans with high occupational exposure to food and primary care patients in Switzerland.

**Methods:**

Stool samples from 1091 healthy individuals and fecal swabs from 53 primary care patients were screened for polymyxin-resistant *Enterobacteriaceae* using LB agar containing 4 mg/L colistin. Minimal inhibitory concentrations (MICs) of colistin were determined for non-intrinsic colistin-resistant isolates. Isolates were screened by PCR for the presence of *mcr-1* and *mcr-2* genes.

**Results:**

The fecal carriage rate of colistin resistant (MIC value >2 mg/l) *Enterobacteriaceae* was 1.5% for healthy people and 3.8% for primary care patients. Isolates included *Hafnia alvei* (*n* = 9), *Escherichia coli* (*n* = 3), *Enterobacter cloacae* (*n* = 4), *Klebsiella pneumoniae* (*n* = 1) and *Raoultella ornithinolytica* (*n* = 1). None of the isolates harbored the *mcr-1* or *mcr-2* genes.

**Conclusions:**

There is no evidence for the presence of MCR-producers in the fecal flora of healthy people or primary care patients. Therefore, the risk of transfer of *mcr* genes from animals, food or the environment to humans is likely to be low in Switzerland.

## Introduction

Polymyxins are cationic polypeptide antibiotics that interact with the lipopolysaccharides (LPS) and phospholipids in the outer membrane of Gram-negative bacteria [1]. Due to the lack of novel antimicrobials, polymyxin E (colistin), once avoided because of its nephro- and neurotoxicity, has become a last-resort antimicrobial to treat life-threatening infections due to multidrug resistant (MDR) Gram-negative bacteria [2]. However, in the agricultural sector, polymyxins are applied regularly for the treatment of gastrointestinal infections in livestock and their increased use may have promoted the emergence of colistin-resistant bacteria [3]. Acquired resistance to polymyxins in *Enterobacteriaceae* is mainly related to mutations or truncations in the genes encoding the PmrA/PmrB and PhoP/PhoQ two component systems (TCS), or to the expression of acquired *mcr-1* or *mcr-2* genes, which are plasmid-located [4, 5]. In both cases, resistance arises through the modification of lipid A component of the outer membrane by 4-amino-4-deoxy-L-arabinose (L-Ara4N) or phosphoethanolamine (PEtN) [1]. Plasmid-mediated colistin resistance has recently been acknowledged as a major threat to public health [4]. There is evidence that *mcr-1* harboring *Enterobacteriaceae* have been occurring globally in food-producing animals, in food and in humans for several years, often associated with other resistance genes including extended-spectrum ß-lactamases (ESBL) and carbapenemases [6]. Further, the recently identified *mcr-2* gene, which shares 76.7% nucleotide sequence homology with *mcr-1*, has been found to be more prevalent than *mcr-1* in porcine colistin resistant *Escherichia* (*E.) coli* isolates in Belgium [5].

Hence, there is need for continuous surveillance of colistin resistance in *Enterobacteriaceae* in order to reduce the risk to human health. The intestinal microbiota forms a major reservoir of antibiotic resistant bacteria in humans [7], therefore asymptomatic carriage of MCR producers must be taken into account in prevention and control efforts. A recent study showed that 10% of travelers returning from India were fecal carriers of colistin resistant *Enterobacteriaceae*, and had probably acquired such strains via the food chain [8]. This study was conducted in order to (i) assess the occurrence of colistin-resistant *Enterobacteriaceae* in the fecal flora of healthy people with high occupational exposure to food and of primary care patients in Switzerland during the period of June to October 2016, and (ii) determine whether any of the resistant isolates harbored *mcr-1* or *mcr-2*. Because Switzerland is situated at the geographical center of Europe and represents a socioeconomic and demographic intersection of the surrounding countries, this country is ideal for observing temporal-spatial trends in the occurrence of antibiotic resistance in the population of central Europe.

## Materials and methods

In total, 1144 non-duplicate samples were analysed. Stool samples (*n* = 1091) were obtained by the National Centre for Enteropathogenic Bacteria and *Listeria* (NENT) from employees of food-processing companies located throughout Switzerland between July and October 2016, during a yearly routine fecal screening for *Salmonellae*. Fecal swabs (*n* = 53) were obtained from adult primary care patients consulting their general practitioner in a suburban community in the greater area of Zurich, Switzerland, during a period of 2 weeks in September 2016. Informed consent was obtained from each participating patient and the study was approved by the local ethics committee of Zürich (BASEC-Nr. Req-2016-00374).

Stool samples (one loopful each) and swabs were enriched in 5 ml *Enterobacteriaceae* enrichment (EE) broth (BD, Franklin Lakes, NJ, USA) for 24 h at 37 °C. Thereafter, one loopful was streaked onto LB agar plates containing 4 mg/L colistin, 10 mg/L vancomycin and 5 mg/L amphotericin B for selection of colistin-resistant Gram-negative bacteria. Colonies were identified using API ID 32 E (bioMérieux, Marcy l’Etoile, France) or by 16S rRNA or *rpoB* custom sequencing (Microsynth, Balgach, Switzerland). Species with intrinsic resistance to polymyxins (*Serratia marcescens, Proteus* spp., *Providencia* spp. and *Morganella* spp.) were discarded. All other isolates were selected for further analysis.

Determination of the minimum inhibitory concentration (MIC) of colistin was performed by broth microdilution according to the European Committee on Antimicrobial Susceptibility Testing EUCAST (eucast.org). Screening by PCR for *mcr-1* and *mcr-2* was performed as described previously [4, 9] using DNA from *mcr-1* harboring strain OW3E1 [10] and plasmid “Plasmid-MCR2-Positivkontrolle” (P. Keller, personal communication) as positive controls.

## Results and discussion

A total of 62 isolates were obtained from the selective plates. Thereof, 18 were resistant to colistin (MIC >2 mg/L), including *Hafnia (H.) alvei* (*n* = 9), *E. coli* (*n* = 3), *Enterobacter (E.) cloacae* (*n* = 4), *Klebsiella (K.) pneumoniae* (*n* = 1) and *Raoultella (R.) ornithinolytica* (*n* = 1).

*H. alvei* and *E. coli* were identified using API 32 E, *R. ornithinolytica* by 16S rRNA sequencing, *K. pneumoniae* and *E. cloacae* by *rpoB* sequencing. Resistant isolates originated from 16 (1.5%) of the healthy people and 2 (3.8%) of the primary care patients (Table 1). Of the primary care patients, both were male and the median age was 54.5 years. Underlying conditions were diabetes (2/2, 100%) and cardiovascular diseases (2/2, 100%). Both (100%) had had contact with a hospital as outpatients for diagnostic radiography or colonoscopy. Neither of the patients had a recent history of travel or treatment with antimicrobials within 6 months prior to sampling. Neither of the patients’ professional occupations were associated with close contact to agriculture.

**Table 1:** Colistin resistant Enterobacteriaceae isolated from the fecal flora of 1091 healthy humans and 53 primary care patients

Species	No. of isolates from		No. of isolates with MIC of colistin [mg/L] with values of		
	Healthy people	Primary care patients	4	8	64
*H. alvei*	8	1	5	4	0
*E. coli*	3	0	1	2	
*E. cloacae*	3	1		4	
*R. ornithinolytica*	1	0		1	
*K. pneumoniae*	1	0			1

Results of the PCR screening for *mcr-1* and *mcr-2* remained negative for all 62 isolates.

This study presents the first report on the occurrence of colistin-resistant *Enterobacteriaceae* in the fecal flora of healthy humans and primary care patients in Switzerland and Europe. The absence of MCR producers in the fecal flora of healthy people as well as primary care patients is of major epidemiological interest. It indicates that the risk of transfer of *mcr* genes from animals, food or the environment is currently very low in the community, despite the fact that colistin is used for treating infections in livestock. Indeed, there is recent evidence that food-producing animals in Switzerland do not represent an *mcr-1* or *mcr-2* reservoir, thus a risk of transfer from livestock to humans may be excluded [11]. By contrast, 25.8% of (partly imported) poultry meat samples in Swiss retail stores contain *mcr-1* harboring *E. coli* [12]. Despite this potential threat to public health, the risk of transfer of *mcr-1* genes from food to humans appears to be very low, even for individuals working in the food-processing industry, as demonstrated by the data presented here. This finding correlates with previous reports on the low prevalence (<1%) of infections of humans with MCR-producing *Enterobacteriaceae* in Switzerland [9]. MCR producers have so far been detected rarely in stool samples of healthy individuals and reports are currently restricted to Asian countries [13, 14]. Additional studies should be carried out in order to continuously evaluate the dissemination of *mcr* genes among enteric bacteria in animals, food and humans. Finally, fecal carriage of clinically significant bacteria such as *E. coli, E. cloacae* and *K. pneumoniae* with likely non-transmissible colistin resistance may be of concern, should such bacteria acquire multidrug resistance plasmids.

## Conclusions

Currently, plasmid-mediated colistin resistance poses no threat to public health in Switzerland. Regular and updated information on the occurrence of *mcr* genes in bacterial isolates from animals, the food chain and humans at global, national and regional levels is essential to anticipate future trends in the prevalence and dissemination of plasmid-mediated colistin resistance.

## Abbreviations

EE: *Enterobacteriaceae* enrichment
ESBL: Extended-spectrum ß-lactamases
EUCAST: European Committee on Antimicrobial Susceptibility Testing
L-Ara4N: 4-amino-4-deoxy-L-arabinose
LPS: Lipopolysaccharides
MDR: Multidrug resistant
MICs: Minimal inhibitory concentrations
NENT: National Centre for Enteropathogenic Bacteria and *Listeria*
PEtN: Phosphoethanolamine
TCS: Two component system
